# Computed tomography findings of hepatic veno-occlusive disease caused by *Sedum aizoon* with histopathological correlation

**DOI:** 10.1590/1414-431X20154563

**Published:** 2015-11-23

**Authors:** H. Shao, H. Z. Chen, J. S. Zhu, B. Ruan, Z. Q. Zhang, X. Lin, M. F. Gan

**Affiliations:** 1 Department of Infectious Diseases, Taizhou Hospital Affiliated to Wenzhou Medical College, Linhai, China; 2 State Key Laboratory for Diagnosis and Treatment of Infectious Disease, the First Affiliated Hospital, College of Medicine, Zhejiang University, Hangzhou, China; 3 Collaborative Innovation Center for Diagnosis and Treatment of Infectious Diseases, Hangzhou, China; 4 Department of Infectious Disease, Xianju Hospital of Traditional Chinese Medicine, Xianju, China

**Keywords:** Hepatic veno-occlusive disease, *Sedum aizoon*, Drug-induced liver disease, Computed tomography

## Abstract

This study investigated the value of computed tomography (CT) in the diagnosis and treatment of hepatic veno-occlusive disease (HVOD) caused by *Sedum aizoon* (SA). The clinical manifestations, treatment results, imaging findings, and histological findings of the liver were analyzed in 39 patients with HVOD caused by SA. Hepatomegaly, liver dysfunction, abdominal effusion, and geographic density changes on liver CT scans were found in all 39 patients. The pathological findings of histological liver examination included swelling and point-like necrosis of liver cells, significant expansion and congestion of the sinuses, endothelial swelling, and wall thickening with incomplete lumen occlusion of small liver vessels. CT geographic density changes were confirmed by histological examination of the liver in 18 patients. Sixteen patients with small amounts of ascites that started within 4 weeks of treatment recovered completely or significantly improved after symptomatic and supportive treatment. However, only 43.75% of the patients with larger amounts of ascites improved following symptomatic and supportive treatment. In conclusion, liver CT examination is a valuable, safe, and noninvasive tool for the diagnosis of HVOD caused by SA. In selected cases, liver CT examination may replace liver biopsy and histological analysis.

## Introduction

Hepatic veno-occlusive disease (HVOD), also termed sinusoidal obstruction syndrome, was first reported by Jelliffe in 1954 (1). The disease refers to intrahepatic post-sinusoidal portal hypertension caused by stenosis or occlusion of the veins, which may result from damage to the central veins of the hepatic lobules and the sublobular vein. It is characterized by painful hepatomegaly, jaundice, and ascites (2) and is associated with high mortality (3). The main clinical manifestations include hepatomegaly, hepatalgia, and ascites. Most cases of HVOD are reported in patients who undergo bone marrow transplantation (4,5); in China, however, this disease is also caused by ingestion of *Sedum aizoon* (SA) (6).

SA is widely distributed in Fujian, Jiangsu, Zhejiang, and Henan provinces of China. This herb has been used in folk medicine to stop bleeding without stasis, tranquilize and detoxify patients, and treat pain or trauma, particularly due to hemorrhage (6,7). It has also been included in traditional Chinese medicine standards in Jiangsu and Fujian provinces (8,9). Several compounds have been isolated from ethanolic extracts of SA, such as flavonoids, terpenoids, and alkaloids (9,10), and the hemostatic activity of SA may be related to the presence of gallic acid, vanillic acid, and luteolin (11). In recent years, increasing numbers of cases of HVOD caused by SA have been reported in China (6). However, there are few reports on the clinical diagnosis and treatment of HVOD caused by SA. We herein report 39 cases of HVOD caused by SA and provide a retrospective analysis of the diagnosis and treatment.

## Patients and Methods

This was a retrospective study of clinical information gathered at Taizhou Hospital Affiliated to Wenzhou Medical College. Information was collected on 39 patients hospitalized for HVOD caused by SA (18 males, 21 females) from July 2005 to December 2013. The median patient age was 55 years (range: 33-73 years). All patients had taken SA for either an injury or fracture 2-16 weeks prior to hospitalization (total SA dose: 300-900 g; manner of administration: decoction of fresh SA *po* once a day; median administration time: 12 days [range: 3-21 days]). The exclusion criterion was the presence of other hepatic diseases. After taking SA for 13 days (range: 3-21 days), the patients experienced clinical manifestations such as upper right quadrant abdominal pain, hepatomegaly, liver dysfunction, and ascites.

The diagnosis was made according to the following criteria (12): i) increased serum bilirubin level (≥34.2 µmol/L) without other explainable causes, and ii) hepatomegaly with pain and ascites and an unexplainable weight gain of ≥2%, or iii) small hepatic vein obstruction, hepatic venous lumen eccentric stenosis and induration, or iv) sinusoidal fibrosis and hepatocellular necrosis in zone three found by percutaneous liver biopsy. Clinical data, laboratory test results, and imaging data were collected for all patients.

Written informed consent to participate was obtained from all patients. This study was approved by the Ethics Committee of Taizhou Hospital Affiliated to Wenzhou Medical College (#2005-037).

Descriptive statistical analyses were performed using SPSS 15.0 (SPSS Inc., USA). P<0.05 indicated statistical significance. Variables are reported as median and range.

## Results

### Clinical manifestations of HVOD

Most patients exhibited upper right quadrant abdominal pain, hepatomegaly, and ascites together with nausea, vomiting, malaise, and other gastrointestinal symptoms. Sixteen patients undergoing routine examination of the ascites were found to have a transudate, and 17 patients had a fever with a body temperature between 37.6°C and 38.3°C, which returned to the normal range after 2-4 days. Renal function test results were normal for all patients, although 30 patients had a reduction in urine volume in the 24 h following admittance (20 of these patients had urine volumes of <500 mL per 24 h, and one patient had urine volume of <50 mL per 24 h) (Table 1). Liver function test results showed that all patients had increased alkaline phosphatase and hyaluronic acid levels. The median alanine aminotransferase (ALT) value was 376 U/L (range: 35-500 U/L); 28 patients had an ALT level within the upper limit of normal, and 9 had an ALT level of >4 times the upper limit of normal. Thirty-eight patients had a median total bilirubin level of 67.3 µmol/L (range: 34.2-85.5 µmol/L), and 1 patient had a total bilirubin level of 386.5 µmol/L. The median albumin level was 33 g/L (range: 23-37 g/L); 8 patients had an albumin level of <30 g/L. All patients had a prothrombin time of >14.5 s; the prothrombin time was prolonged by 2 s in 10 patients and by 4 s in 6 patients (Table 2).

**Table:**
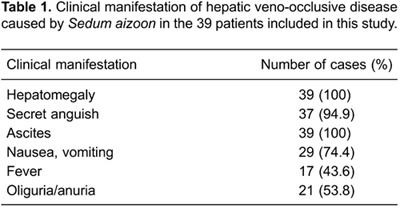

**Table:**
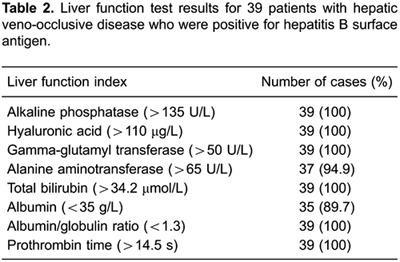

### B-mode ultrasonographic findings of liver and gallbladder

All 39 patients had nonuniformly increased echogenicity of the liver parenchyma on ultrasound examination, and patchy hypoechoic areas were found in 21 cases. Ascites was found in all patients; the median portal vein diameter was 1.2 cm (range: 1.1-1.3 cm), and the median thickness of the spleen was 4.1 cm (range: 3.5-4.8 cm). The circulation of blood in the inferior vena cava, portal vein, and hepatic vein was smooth; however, blood circulation in the hepatic artery was slow.

### CT examination of the liver

**Figure 1:**
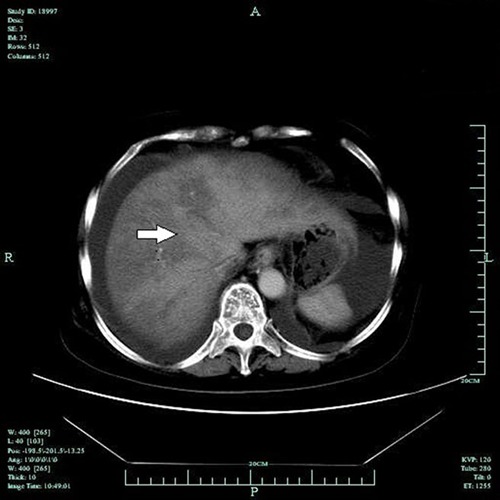
Unenhanced CT image of the liver of a patient with hepatic veno-occlusive disease. The arrow shows the lower geographic liver section, which is slender.

**Figure 2:**
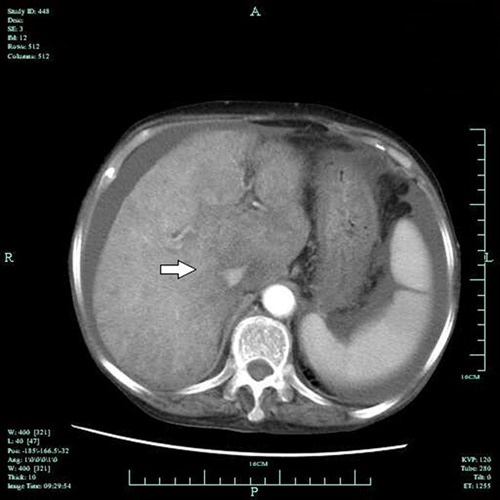
Unenhanced CT image of the inferior vena cava in the liver section of a patient with hepatic veno-occlusive disease. The arrow shows the inferior vena cava with non-homogeneous density.

**Figure 3:**
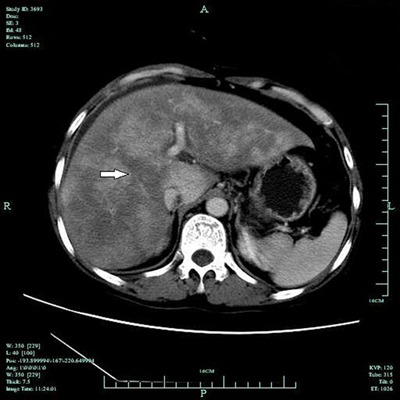
Enhanced liver CT image in the portal venous phase. The arrow shows the density change of the geographic liver section.

All 39 patients showed hepatomegaly and diffuse, heterogeneous density of the liver parenchyma with scattered hypodense areas. Thirty-six patients showed heterogeneous density with geographic areas of hypodensity, and three patients showed diffuse hypodensity of the whole parenchyma (Figure 1). The median CT value was 46 HU (range: 42-49 HU). An enlarged spleen with preserved density was found in 33 patients. The arterial phase on dynamic contrast-enhanced CT showed a disordered network of intrahepatic vasculature; the hepatic artery was slightly enlarged, and the hepatic veins were barely visible. The inferior vena cava in the liver section was found to be slender, and the geographic density of the liver was enhanced (Figure 2). The portal phase detected geographic areas of hypodensity in all 39 patients (Figure 3).

### Pathological examination of liver biopsy

**Figure 4:**
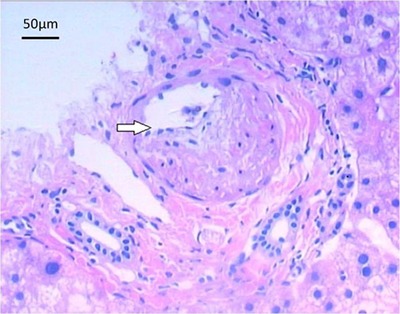
Microscopic image of a section of the liver taken following biopsy. The arrow shows the enhanced thickness of the hepatic portal vein wall in the portal area. Note that the venous lumen is not fully occluded. Hematoxylin-eosin staining, 100×.

Eighteen patients with small amounts of ascites (negative shifting dullness in the abdomen) were selected for percutaneous liver biopsy with examination using hematoxylin-eosin staining. Microscopic evaluation showed swelling of the liver cells, spotty necrosis, endothelial swelling and thickened walls of the hepatic vein, and significantly expanded sinusoids with congestion. The lumen of the hepatic vein was not fully occluded, and it contained no thrombosis. There was also no distinctive sign of fibrosis. The pathological diagnosis was consistent with the manifestations of acute HVOD (Figure 4).

### Therapeutic effect

All 39 patients stopped taking SA and were treated with low-molecular-weight heparin, reduced glutathione, human serum albumin, and diuretics for 7-21 days. Seven patients fully recovered, and 19 improved. However, 13 did not improve following treatment, and 1 died. Sixteen patients with small amounts of ascites and an onset of symptoms within 4 weeks of treatment fully recovered or significantly improved after symptomatic and supportive treatment. Of 4 patients with small amounts of ascites and an onset of symptoms more than 4 weeks prior to treatment, 3 either fully recovered or improved; however, 1 patient had increased ascites after treatment. The rate of recovery or significant improvement among patients with larger amounts of ascites (positive shifting dullness in the abdomen) was only 43.75% (7 of 16 patients).

## Discussion

Hill et al. (13) first reported the risk of HVOD caused by rooibos tea containing pyrrolidine alkaloids (PAs) in Jamaica and Barbados. In the past 20 years, HVOD has been reported to be related to anticancer chemotherapy. According to some reports, the incidence of HVOD following allogeneic bone marrow transplantation is 8.9%, compared with 3.1% following autologous marrow transplantation (14). HVOD is also associated with other agents, such as busulfan, cyclophosphamide, deticene, and azathioprine (15-18).

There are two main causes of HVOD: i) cytotoxic or immunosuppressive agents, such as cyclophosphamide and carmustine, and ii) agents containing PAs, such as Qianliguang, SA, and *Crotalaria* (19). DeLeve et al. (20) used PA-containing fodder to feed mice and established animal models of HVOD. Zuckerman et al. (21) used colorimetry and mass spectrometry to analyze traditional medicines containing PAs and then tested the toxicity of SA extracts in liver cell cultures. They found that the liver toxicity of the extracts was dose-dependent. Meanwhile, Gao et al. (22) found that there are more than 5 types of PAs in SA.

The clinical features of HVOD have been subdivided into 3 clinical stages: i) acute stage, in which hepatomegaly develops suddenly over 5-10 days with ascites; ii) subacute stage, in which firm hepatomegaly with or without ascites may occur spontaneously or after the acute stage and may subside or become chronic; and iii) chronic stage, in which cirrhosis occurs (23-25).

In general, the onset of symptoms in the 39 patients with HVOD who had taken SA medicinally after accidental injury or fracture in the present study occurred approximately 14 days after SA administration. In the acute phase, most patients first showed upper right quadrant pain, followed by abdominal distension and ascites, hepatomegaly, and liver dysfunction. These symptoms were accompanied by fatigue, nausea, vomiting, and other gastrointestinal symptoms. The severity of symptoms depended on the dose of SA.

The diagnosis of HVOD is based on the patient’s history of illness, clinical manifestations, imaging results, and pathological features. Percutaneous liver biopsy is the gold standard for HVOD diagnosis, but there are associated risks and the possibility of trauma. The histopathological features of HVOD include hepatic vein occlusion, hepatic venous lumen eccentric stenosis and induration, sinusoidal fibrosis, and liver cell necrosis in zone three. Pathological manifestations in the acute phase include hepatocellular necrosis, endothelial swelling of the hepatic vein, thickening of the hepatic vein walls, and significant expansion and congestion of the sinuses. However, total occlusion of the lumen is not apparent. In the present study, such pathological manifestations were found in 15 patients in the acute phase; these patients had small amounts of ascites and were selected for percutaneous liver biopsy. The pathological manifestations among patients in the subacute and chronic phases included central lobular fibrosis and non-portal liver cirrhosis, the latter particularly in the chronic phase.

Few reports have described the liver CT imaging findings for the diagnosis of HVOD. The normal CT liver density reportedly ranges from 45 to 55 HU (26). In the present study, CT scanning showed that all 39 patients had ascites and decreased, nonuniform hepatic parenchymal density. Of these, 36 displayed nonuniformly distributed enhanced geographic areas of low density in the liver parenchyma, with median CT value of 46 HU; the remaining three displayed diffuse hypodensity of the hepatic parenchyma. The arterial phase depicted disarrangement of the intrahepatic network vasculature along with a subtle increase in the hepatic artery and fading of the hepatic veins, while the inferior vena cava was slender. In the portal vein phase, the geographic areas of hypodensity were more conspicuous in all 39 patients. These CT findings were confirmed by percutaneous liver biopsy in 18 patients. We found that the CT imaging findings in patients with HVOD were closely correlated with the histologic changes in the liver parenchyma. The geographic areas of hypodensity on CT scans correspond with necrosis of the hepatic parenchymal cells in patients with HVOD (27). PAs can damage the Na^+^-K^+^pump on the liver cell membrane, resulting in cellular swelling, necrosis, and hepatomegaly (27,28). The heterogeneous liver density was in accordance with the inhomogeneous enhancement on enhanced CT, which was more obvious in the portal venous phase. Dumont et al. (29) reported that CT enhancement changes in patients with HVOD were related to blood stasis in hepatic sinusoids. The slow blood flow in hepatic sinusoids leads to decreased enhancement of the injured hepatic parenchyma, forming geographic areas of hypodensity. Therefore, we regarded the geographic areas of hypodensity in the liver as a characteristic manifestation of HVOD. For patients with large amounts of ascites and who were not suitable candidates for percutaneous liver biopsy, diagnostic liver CT examination was adopted as an alternative to HVOD. This allowed differentiation from hepatic vein thrombosis and Budd-Chiari syndrome.

Therapy for HVOD is a complex issue. Lee et al. (30) found that low-molecular-weight heparin alone or in combination with recombinant tissue plasminogen activator (rt-PA) had an effect on severe HVOD. Richardson et al. (31) found that defibrotide was effective for severe HVOD without obvious adverse reactions. Liver transplantation is also a feasible choice for the treatment of HVOD. However, a small-sample study showed that the survival rate following liver transplantation reached 30% in a clinical setting (32). Our results indicate that most patients in the acute and subacute phases improved after stopping SA administration and starting symptomatic treatment. Further analysis showed that all 16 patients with small amounts of ascites and an onset of symptoms within 4 weeks of admittance fully recovered or significantly improved after symptomatic and supportive treatment. Only 43.5% of patients with larger amounts of ascites improved.

In summary, our study indicates that liver CT examination is a safe technique for the early diagnosis of HVOD caused by SA and can replace histological examination of the liver when clinical data and imaging findings are typical. The curative effect of treatment improved when patients were admitted soon after developing symptoms and for those that had a small amount of ascites. Physicians in areas where SA is widely used should intensify their education to improve their knowledge about the risks of HVOD. They should also train in the diagnosis of HVOD obtained by liver CT and other examinations and consider that symptomatic and supportive treatment should be given as soon as possible to improve the recovery rate of patients with HVOD due to SA.
